# Identification and Characterization Analysis of Transient Receptor Potential Mucolipin Protein of *Laodelphax striatellus* Fallén

**DOI:** 10.3390/insects12121107

**Published:** 2021-12-12

**Authors:** Haitao Wang, Yan Dong, Baijie Wan, Yinghua Ji, Qiufang Xu

**Affiliations:** 1 Key Laboratory of Food Quality and Safety of Jiangsu Province, Institute of Plant Protection, Jiangsu Academy of Agricultural Sciences, Nanjing 210014, China; dongy19870330@163.com (Y.D.); jiyinghua@jaas.ac.cn (Y.J.); 2 Institute of Agricultural Sciences in Jiangsu Coastal Area, Yancheng 224002, China; wanbaijie@163.com; 3 Institute of Life Sciences, Jiangsu University, Zhenjiang 212013, China

**Keywords:** *Laodelphax striatellus*, TRPML, expression profile, subcellular localization, lipid binding

## Abstract

**Simple Summary:**

The small brown planthopper *Laodelphax striatellus* is a destructive pest of rice, maize and wheat crops in Asia, causing damage by directly sucking phloem sap and transmitting plant viruses, and thus seriously impacting crops yields. Transient receptor potential mucolipin (TRPML) protein plays a vital role in Ca^2+^ ions release, resulting in membrane trafficking, autophagy and ion homeostasis; however, till now, we have learned little about the TRPML protein of agricultural pests. In this study, we first identified the TRPML of *L*. *striatellus*. We analyzed not only the gene evolutionary features and expression profiles but also clarified the protein subcellular localization and lipid-binding characteristics of Ls-TRPML. We found that TRPML is evolutionarily conserved among agricultural pests. Ls-TRPML is predominately expressed in *L. striatellus* ovary. Moreover, we found that Ls-TRPML localizes in the nuclear membrane in *Spodoptera frugiperda* cells and the intestine and ovary of *L. striatellus*. The binding of Ls-TRPML with lipids was detected by lipid-binding assay, indicating the potential role of Ls-TRPML in lipid interaction. Thus, our findings first helped us analyze the gene characterization of Ls-TRPML and then identify the binding of Ls-TRPML with lipids; our findings will broaden our understanding of TRPML’s roles in agricultural pests.

**Abstract:**

Transient receptor potential mucolipin (TRPML) protein in flies plays a pivotal role in Ca^2+^ ions release, resulting in membrane trafficking, autophagy and ion homeostasis. However, to date, the characterization of TRPML in agricultural pests remains unknown. Here, we firstly reported the TRPML of a destructive pest of gramineous crops, *Laodelphax striatellus*. The *L*. *striatellus* TRPML (Ls-TRPML) has a 1818 bp open reading frame, encoding 605 amino acid. TRPML in agricultural pests is evolutionarily conserved, and the expression of Ls-TRPML is predominately higher in the ovary than in other organs of *L*. *striatellus* at the transcript and protein level. The Bac–Bac system showed that Ls-TRPML localized in the plasma membrane, nuclear membrane and nucleus and co-localized with lysosome in *Spodoptera frugiperda* cells. The immunofluorescence microscopy analysis showed that Ls-TRPML localized in the cytoplasm and around the nuclei of the intestine cells or ovary follicular cells of *L*. *striatellus*. The results from the lipid-binding assay revealed that Ls-TRPML strongly bound to phosphatidylinositol-3,5-bisphosphate, as compared with other phosphoinositides. Overall, our results helped is identify and characterize the TRPML protein of *L*. *striatellus*, shedding light on the function of TRPML in multiple cellular processes in agricultural pests.

## 1. Introduction

Members of the transient receptor potential (TRP) family are composed of TRPC (canonical), TRPV (vanilloid), TRPM (melastatin), TRPN (nompc), TRPP (polycystin), TRPA (ankyrin) and TRPML (mucolipin); all of the proteins are responsible for Ca^2+^ permeable nonselective cation channels that bear structural similarities to *Drosophila* TRP [1]. Based on the similarity to *Drosophila* TRP, TRPC, TRPV, TRPM and TRPA are classified as Group 1 TRP channels. The Group 2 TRP channels, including TRPP and TRPML, have distal relevance to *Drosophila* TRP [2]. Currently, the most well-defined TRP functions serve as cellular sensors for detecting an array of environmental stimuli, including temperature, mechanical forces and pain [2,3,4]. The TRPML channels function in the endocytic pathway, which sets them apart from other TRP channels, and they are the only cation channels known so far to be localized and function in the cellular digestive tract [5]. Thus, exploring TRPML function will lead to better a understanding of the ion channel’s role in the endocytic pathway and the integrative function of the cells.

In vertebrates, the mucolipin subfamily of transient receptor potential mucolipin (TRPML) proteins consist of three members, TRPML1, TRPML2 and TRPML3 [6]. In contrast to vertebrates, members of the TRPML subfamily are conserved and encode only a single TRPML in *Caenorhabditis elegans* and flies [1]. *Drosophila melanogaster* TRPML (Dl-TRPML) more closely resembles mammalian TRPML1 than TRPML2 or TRPML3 [7,8], and it is ubiquitously expressed in every tissue and cell type and localized in endolysosomes and plasma membranes, with the N- and C-termini facing the cytosol [6,7].

The TRPMLs in endosomes or lysosomes are non-selective cation channels that conduct Ca^2+^ and monovalent cation currents from the lumen to the cytoplasm [9,10]. As the principal Ca^2+^ release channel in the lysosome, upon activation, TRPML may mediate the release of Ca^2+^ ions. The phosphoinositides are important signal molecules to regulate the membrane trafficking by activating their downstream effectors [11]. Some studies reported that phosphatidylinositol-3,5-bisphosphate [PI(3,5)P_2_] localizes on endolysosomes and plasma membrane and directly activates TRPML to control the membrane trafficking [7,12]. The exogenous oxidants or increasing mitochondrial reactive oxygen species (ROS) levels directly and specifically activate TRPML1 and induce the release of Ca^2+^, resulting in autophagy biogenesis [13,14,15]. Disruption of the only *TRPML* gene of flies resulted in defective autophagy, due to the diminished fusion of late endosomes with lysosomes, accumulation of apoptotic cells, oxidative stress, mitochondrial dysfunction and massive neurodegeneration [16]. Recent research revealed that TRPML also controls the actomyosin contractility and couples migration to phagocytosis in fly macrophages [17].

The *C. elegans* functional *TRPML1* ortholog, *cpu-5*, regulates lysosome biogenesis, and the mutant of *cup-5* results in the large vacuoles accumulated in endosomes and lysosomes and general developmental defects [18,19,20,21,22]. Additionally, the *cup-5* of *C. elegans* is required for the proteolytic degradation in autolysosomes [23]. Thus, the TRPML of flies and worms plays an important role in multiple cellular processes; it is especially involved in the release of Ca^2+^ and acts as a key regulator to manipulate the autophagy pathway. However, to date, the TRPML function in agricultural pest insects remains unknown.

The small brown planthopper *Laodelphax striatellus* Fallén is a destructive pest of rice, maize and wheat crops in Asia, causing damage by directly sucking phloem sap and transmitting plant viruses, such as rice stripe virus, rice black streaked dwarf virus and barley yellow striate mosaic virus [24,25,26]. Although previous studies have investigated TRPML adequately in mammals, flies and worms, the TRPML protein of agricultural pest remains unknown. 

Here, we cloned and analyzed the sequence of *Ls-TRPML* and found that TRPML is evolutionarily conserved among agricultural pests. We found that Ls-TRPML is predominately expressed in the ovary of *L. striatellus*. Moreover, we observed the subcellular localization of Ls-TRPML in *Spodoptera frugiperda* (Sf9) cells and the intestine and ovary of *L. striatellus*. Ls-TRPML binds to PI(3,5)P_2_ strongly, indicating that Ls-TRPML is a potential effector of PI(3,5)P_2_. To our knowledge, we first analyzed the characterization of TRPML in *L. striatellus* and identified the binding of Ls-TRPML with phosphoinositides; this will broaden our understanding of TRPML’s roles in agricultural pests.

## 2. Materials and Methods

### 2.1. Insects and Cells

*L. striatellus*, a small brown planthopper, was originally collected from Haian in Jiangsu Province in Eastern China; the non-rice viruses that infected the rice planthopper colony were screened and reared on rice seedlings (Wuyujing No.3) in a controlled environment, at 26 °C, with 55 ± 5% humidity and a photoperiod (16 h light: 8 h dark). 

*Spodoptera frugiperda* (Sf9) cells (Thermo) were cultured in Sf900™ III SFM (12658-019) supplied with 2% bovine serum at 28 °C in a non-humidified air-regulated non-CO_2_ atmosphere.

### 2.2. Bioinformatics

The amino acid (aa) sequence of *Nilaparvata lugens* TRPML (XP_039298120) was used as a query to search the *L. striatellus* genome (GCA_017141395.1). We obtained the TRPML mRNA sequence and cloned, sequenced the open reading frame (ORF), then submitted the ORF sequence to the NCBI GenBank (MZ476564). The functional modules of Ls-TRPML was predicted by using InterPro (online website: https://www.ebi.ac.uk/interpro/, accessed on 11 December 2021) and Smart (online website: http://smart.embl-heidelberg.de/, accessed on 11 December 2021). The signal peptide of Ls-TRPML was predicted by using NovoPro (online website: https://novopro.cn/tools/signalp, accessed on 11 December 2021). The nuclear localization signal of Ls-TRPML was predicted by using the cNLS Mapper online website (http://nls-mapper.iab.keio.ac.jp/cgi-bin/NLS_Mapper_form.cgi, accessed on 11 December 2021). The amino acid sequences of TRPML or Mucolipin of the agricultural pest insects and *Drosophila melanogaster* were obtained from the NCBI database. The phylogenetic tree was built with the neighbor-joining method, using MEGA7 software, based on the alignment of the sequences determined, using ClustalW. The boot-strap consensus tree was inferred from 5000 replicates.

### 2.3. RNA Extraction and RT-qPCR Assay

The total RNA of pools (50 *L. striatellus*) of second-to-fifth instar nymphs, female and male adults and various organs (central nervous system, salivary gland, intestine, ovary and testis) dissected with tweezers under an optical microscope from 100 *L. striatellus* adults were extracted by RNAiso plus (Takara), according to the manufacturer’s instructions. The cDNA was reverse-transcribed from 1 μg extracted total RNA with PrimeScript™ RT reagent kit with gDNA Eraser (Takara), following the manufacturer’s instructions. RT-qPCR was conducted by using IQ™ 5 multicolor real-time PCR detection system (BIO-RAD) with TB Green Premix Ex Taq reagent (Takara). The relative gene expression was normalized to an internal control gene, *alpha tubulin* (primers listed in Table S1), as we described previously [27], and calculated by 2^−ΔΔCT^ (cycle threshold) method. Each experiment contained three independent biological and three technical replications.

### 2.4. Baculovirus Expression of Ls-TRPML in Sf9 Cells

A recombinant baculovirus expression system was used to investigate the localization of Ls-TRPML expressed in Sf9 cells, according to the manufacturer’s instructions. Briefly, *Ls-TRPML* fused with a 6× His tag (Ls-TRPML-His) (primers listed in Table S1) was cloned and then inserted into a pFastBac1 vector. The pFastBac1-Ls-TRPML-His plasmid was transformed into *Escherichia coli* DH10 Bac cells (Thermo) to prepare the recombinant bacmids. Then the recombinant bacmids DNA were transfected into Sf9 cells, using Lipofectamine 3000 reagent (Thermo) according to the manufacturer’s instructions. The culture supernatant was collected at 3 days after the recombinant bacmids transfected. After 30 h of incubation with the Ls-TRPML-His recombinant baculovirus (culture supernatant), the infected Sf9 cells were analyzed by immunofluorescence microscopy.

### 2.5. Antibody Preparation

Rabbit antibody anti-Ls-TRPML peptides KGWDPTREVSSYPPC were prepared by GenScript (Nanjing, China).

### 2.6. Immunofluorescence Microscopy

The intestines and ovaries of adult *L. striatellus* were dissected on iced 1× PBS (pH 7.4) (Sangon Biotech, Shanghai, China) with tweezers, under an optical microscope; fixed with 2% PFA for 2 h; and then permeabilized with 2% Triton X-100 for 30 min. The organs were immunolabeled with Ls-TRPML-specific peptide rabbit antibody (produced by GenScript, 1:200) for 1 h; then they were rinsed with 1 × PBS for 3 times and immunolabeled with goat anti-rabbit IgG H&L (Alexa Fluor^®^ 594) (Abcam, 1:200) for 1 h at room temperature. At last, the *L. striatellus* organs were dyed with DAPI (C1005, Beyotime Biotec.) for 10 min at room temperature. The Ls-TRPML antibody unlabeled organs of *L. striatellus* were set as the control. All samples were analyzed with LSM 710 (ZEISS, Jena, Germany) for confocal microscopy. 

For baculovirus-infected Sf9 cells, the cells were rinsed with 1× PBS for 3 times, fixed with 2% PFA for 30 min and then permeabilized with 0.2% Triton X-100 for 15 min. The cells were immunolabeled with 6× His-tag antibody (Thermo, MA1-21315-A555, 1:100) for 1 h, then rinsed and dyed with DAPI for 5 min at room temperature. The cells directly incubated with LysoTracker™ Deep Red (Invitrogen, 1:200) for 30 min at dark environment. The LysoTracker-treated cells were rinsed, fixed, permeabilized and immunolabeled with 6× His-tag antibody (Thermo) for 1 h; then they were rinsed and dyed with DAPI for 5 min at room temperature. All samples were analyzed with LSM 710 (ZEISS, Jena, Germany).

### 2.7. Plasmid Construction

The nucleotide sequence of *Ls-TRPML* open reading frame of *L. striatellus* was amplified by PrimeSTAR^®^ Max DNA Polymerase (Takara) and cloned into pEASY-Blunt zero vector (TransGen Biotech) for sequencing. The *Ls-TRPML* gene was amplified by the primers listed in Table S1 and constructed into pMal-C2X for protein expression. The positive clones were sequenced and detected with the restriction enzyme (Takara) by double digestion, according to the manufacturer’s instructions.

### 2.8. Protein Expression and Purification

The *E*. *coli* BL21 (DE3) *Rosetta* cells containing expression plasmids were grown at 37 °C to an optical density at 600 nm (OD_600_) of 0.6–0.8 and induced with 0.4 mM IPTG at 16 °C for 16 h. The induced cells were centrifuged at 12,000 rpm, 4 °C for 10 min, and then sonicated in lysis buffer (50 mM Tris-Cl, 200 mM NaCl, 5% glycerol, 5 mM DTT and 1% Triton X-100, pH 8.0). The sonicated cells were centrifuged at 15,000× *g*, 4 °C for 30 min. Then the supernatant was filtered with 0.45 μm filter before incubation with amylose column for maltose binding protein (MBP)-tagged protein at 4 °C for 1 h. MBP-tagged proteins were washed with reduced salt (20 mM Tris-HCl, 25 mM NaCl, 1 mM EDTA, 10 mM β-mercaptoethanol, pH 8.0) and eluted with maltose elution buffer (3.6 mg/mL maltose in reduced salt). The purified proteins were store at −20 °C for further use.

### 2.9. SDS–PAGE and Western Blot Assay

The organs of 300 *L. striatellus* were dissected on iced 1× PBS buffer with tweezers, under an optical microscope, and immediately lysed with RIPA (radio immunoprecipitation assay) lysis buffer (P0013B, Beyotime Biotec.). The induced cells were centrifuged at 12,000 rpm for 2 min. All samples were suspended with 5× loading buffer (2.5 mM Tris-HCl, 0.1% *w*/*v* SDS, 0.005% *w*/*v* BPB, 0.4% *w*/*v* glycerin, 500 mM DTT, pH 6.8), boiled for 10 min and then detected with SDS–polyacrylamide gel electrophoresis (PAGE). The gels were then stained with Coomassie Brilliant Blue R250. After SDS–PAGE, the proteins were transferred to PVDF membranes by eBlotTM L1 (Genscript) and incubated with Ls-TRPML polypeptide primary antibody (produced by Genscript, 1:2000) or MBP Rabbit Monoclonal Antibody antibody (AF1225, Beyotime Biotec., 1:1000), followed with HRP conjugated secondary antibody (Beyotime Biotec., 1:1000). The blotted membranes were visualized by using a chemiluminescence gel imaging system.

### 2.10. Lipid Binding Assay

The PIP strips (P-6001-2, Echelon Biosciences) were blocked with 3% fatty acid-free bovine serum albumin (BSA) (A602448, Sangon Biotech, Shanghai, China) (non-esterified fatty acid < 0.01%) in 1× TBS (20 mM Tris, 150 mM NaCl, pH 8.0) for 1 h, and then the strips (nitrocellulose membrane) were incubated with MBP-Ls-TRPML (final concentration: 5 μg/mL) or MBP (final concentration: 5 μg/mL) protein for 4 h at room temperature, respectively. The strip was thoroughly washed with TBS buffer and then incubated with specific MBP antibody (AF1225, Beyotime Biotec., 1:1000) for 3 h, respectively, followed by incubating with HRP-conjugated secondary antibody (Beyotime Biotec., 1:1000) for 1 h at room temperature. The strips were then analyzed by using an chemiluminescence gel imaging system. Each lipid-binding assay was repeated at least 3 times.

### 2.11. Statistical Analysis

Data were analyzed by using the SPSS software (version 19.0). One-way ANOVA, followed by Tukey’s test, was applied for multiple comparisons.

## 3. Results

### 3.1. Identification of TRPML in L. striatellus

The amino acid (aa) sequence of *Nilaparvata lugens* TRPML (AOR81477.1, ~66.33 kilodalton, KDa) was used as a query to search the *L*. *striatellus* genome (GCA_017141395.1). We amplified, cloned and sequenced the open reading frame of *Ls-TRPML* (1818 bp) (Figure S1B) and then submitted the sequence to NCBI GenBank (MZ476564). TRPML in *Caenorhabditis elegans* and flies contains five or six transmembrane regions [2]. To analyze the potential functional modules of Ls-TRPML, we firstly predicted Ls-TRPML domains by using the Smart online website and found that Ls-TRPML encodes 605 amino acids protein (~66.55 KDa) and contains five transmembrane regions, from 304 to 522 aa (Figure S1A,C). Additionally, we analyzed the Ls-TRPML protein by InterPro online website and found that Ls-TRPML is one canonical member of Mucolipin (TRPML) family, contains one transmembrane region (304–326 aa) and a Polycystic kidney diseases (PKD) channel domain (335–528 aa) (Figure S1D), indicating that Ls-TRPML is one member of TRPML and the functional modules are similar to the TRPML of flies and worms.

**Figure 1:**
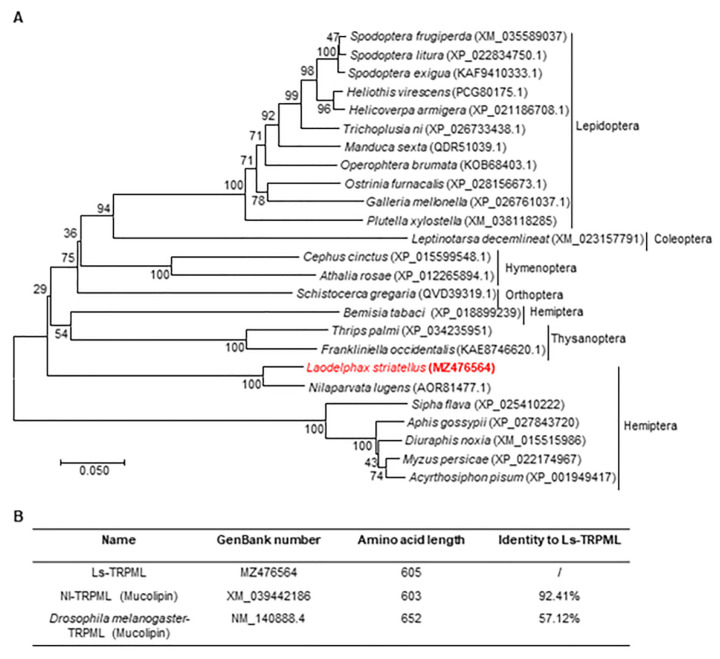
Identification of Ls-TRPML. (**A**) Evolutionary phylogenetic tree of agricultural pest TRPML proteins was constructed by using the neighbor-joining (NJ) method based on the alignment of TRPML (Mucolipin) protein sequences obtained from NCBI database. Bootstrap values of 5000 replicates are indicated at the branch nodes. Tree is drawn to scale, with branch lengths in the same units as those of the evolutionary distances used to infer the phylogenetic tree. Evolutionary distances were computed by using the Poisson correction method and are in the units of the number of amino acid substitutions per site. Evolutionary analyses were conducted in MEGA7. (**B**) Percentages of amino acid identities between Ls-TRPML and *Nilaparvata lugens* TRPML (Nl-TRPML) or *Drosophila melanogaster* TRPML (Dm-TRPML).

To analyze whether TRPML is conserved in agricultural pests, we constructed a phylogenetic tree of the TRPML protein sequences of 25 agricultural pest insects from Lepidoptera, Coleoptera, Hymenoptera, Orthoptera, Hemiptera, Thysanoptera and Hemiptera. The result showed that TRPML of *Leptinotarsa decemlineat* from the Coleoptera has a close relation with the Lepidoptera TRPML protein sequence (Figure 1A). The TRPML protein is evolutionarily conserved in Lepidoptera; interestingly, the *Plutella xylostella* has a relative distal relevance to other insects TRPML of Lepidoptera (Figure 1A). What is more, the *Bemisia tabaci* from Hemiptera has a close relation with thrips from the Thysanoptera (Figure 1A). We found that the TRPML amino acids of aphids, including *Sipha flava*, *Aphis gossypii*, *Diuraphis noxia*, *Myzus persicae* and *Acyrthosiphon pisum*, have close genetic relationship, the *Sipha flava* have a relative distal relevance to the other four aphids TRPML (Figure 1A). Additionally, we found that the *D. melanogaster* TRPML protein sequence has a 57.12% similarity with Ls-TRPML, and Nl-TRPML protein sequence has a 92.41% similarity with Ls-TRPML (Figure 1), indicating that TRPML is evolutionarily conserved in insects. All of these data suggested that TRPML is highly conserved in agricultural pests. In order to analyze the Ls-TRPML property in more detail, we analyzed and found that Ls-TRPML does not contain signal peptide. Moreover, the nuclear localization signals of Ls-TRPML were predicted by using the cNLS Mapper online website, and we found that Ls-TRPML contains a bipartite nuclear localization signal (573–600 aa, KRTVSKLCCWRKPTLTSFVLGRKKTYPP), with a cutoff score of 6 (Figure S1A,B), indicating that Ls-TRPML may play roles in the nuclei of *L. striatellus*.

### 3.2. The Expression Profile Analysis of Ls-TRPML

**Figure 2:**
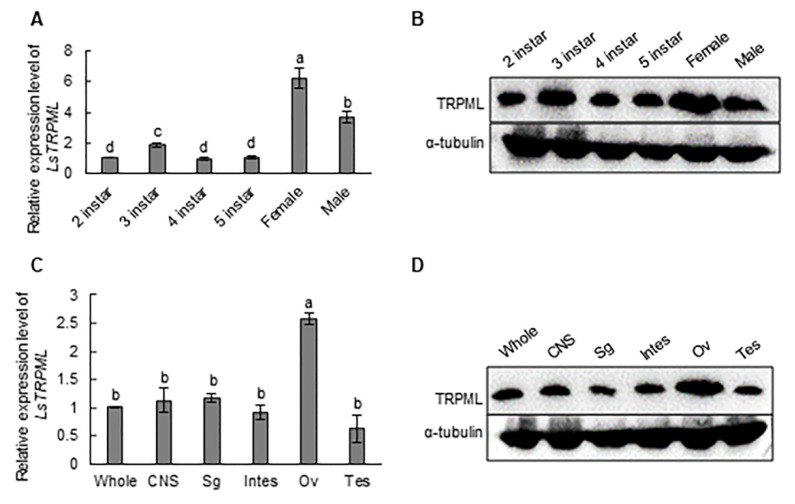
Expression profiles of Ls-TRPML in *L*. *striatellus*. (**A**) Expression of *Ls-TRPML* in different instar, female and male *L*. *striatellus* was detected by RT-qPCR assay. (**B**) Protein accumulation of Ls-TRPML in different instar, female and male *L*. *striatellus* was detected by Western blotting. Alpha tubulin protein was detected as the loading control. (**C**) Transcript level of *Ls-TRPML* in whole insect and indicated different tissues of *L*. *striatellus* was detected by RT-qPCR assay. (**D**) Expression of Ls-TRPML in different organs of *L*. *striatellus* was detected by Western blotting. Alpha tubulin protein was detected as the loading control. Gene expression was normalized to the transcript level of internal control *alpha tubulin* (primers listed in Table S1) gene and estimated by using the 2^−ΔΔCt^ (cycle threshold) method. CNS, central nervous system; Sg, salivary gland; Intes, intestine; Ov, ovary. Multiple comparisons of the means (±SE) were conducted by using a one-way analysis of variance and Tukey’s honest significant difference (HSD) test. Values are the means (±SE) of three biological replicates.

In order to investigate the potential role of Ls-TRPML in the life progress of *L. striatellus*, we analyzed the expression profiles of Ls-TRPML in second instar to fifth instar, female and male adult *L. striatellus*. We found that the transcript level of *Ls-TRPML* in adult *L. striatellus* was notably higher than that in nymphs, and the expression of *Ls-TRPML* in the female rice planthopper was significantly higher than the expression in male *L. striatellus* (Figure 2A), indicating the possible role of Ls-TRPML in females. What is more, we found that the expression of Ls-TRPML in third-instar *L. striatellus* was relative higher than in other instar nymphs *L. striatellus*, and there were no significant differences among second, fourth and fifth instar *L. striatellus* nymphs (Figure 2A). 

To reveal the expression of Ls-TRPML at the protein level, we prepared the rabbit anti-Ls-TRPML polyclonal antibody against Ls-TRPML peptide KGWDPTREVSSYPPC (produced by GenScript) to detect the Ls-TRPML expression in different instars, female and male *L. striatellus*. The Ls-TRPML peptide antibody could detect Ls-TRPML protein specifically (Figure S2). The accumulation of Ls-TRPML in females was significantly higher than that in other instars or male *L. striatellus* (Figure 2B), as was consistent with the transcript levels of Ls-TRPML in *L. striatellus*.

In order to elucidate the expression of Ls-TRPML further, we dissected the organs (central nervous system, salivary gland, intestine, ovary and testis) of adult *L. striatellus* and detected the transcript expression levels of *Ls-TRPML*. We found that the abundance of *Ls-TRPML* in the ovary was significantly higher than that in other tissues, and there were no differences of Ls-TRPML expression in other tissues of *L. striatellus* (Figure 2C). Additionally, Western blotting was performed to detect the protein accumulation of Ls-TRPML in different organs. We found that the expression of Ls-TRPML was notably higher in the ovary than in other organs (Figure 2D). These data suggest the potential role that Ls-TRPML played in the ovary of *L. striatellus*. Taken together, all of the data indicated that Ls-TRPML may play a role in female adult *L. striatellus*.

### 3.3. The Subcellular Localization of Ls-TRPML in Sf9 Cells

**Figure 3:**
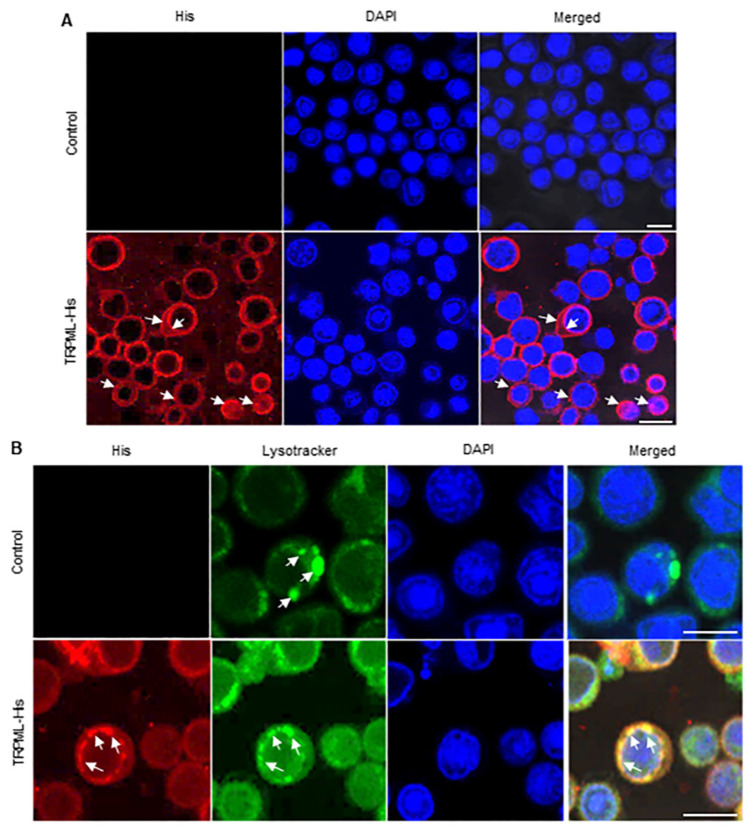
Subcellular localization analysis of Ls-TRPML in Sf9 cells. (**A**) Ls-TRPML-His localization in Sf9 cells. Cells were infected with Ls-TRPML-His baculovirus for 48 h, then rinsed, fixed, permeabilized and dyed with His antibody (red), DAPI (blue). Arrows indicate the localization of Ls-TRPML-His. The uninfected Sf9 cells were introduced as control. Scale bars, 10 μm. (**B**) Ls-TRPML-His co-localized with LysoTracker in Sf9 cells. Cells were infected with Ls-TRPML-His baculovirus for 48 h, then rinsed, incubated with LysoTracker (green), then fixed, permeabilized and immunolabeled with His antibody (red), DAPI (blue), finally examined with confocal microscopy. Arrows note the localization of LysoTracker and Ls-TRPML-His. Uninfected Sf9 cells were introduced as control. Scale bars, 10 μm.

To further uncover the localization of Ls-TRPML, we first introduced the baculovirus expression system to investigate the Ls-TRPML subcellular localization in *Spodoptera frugiperda* (Sf9) cells. We found that the recombinant bacmids of Ls-TRPML-His (presented as red) were expressed in the plasma membrane, nuclear membrane, nucleus, and some aggregated and formed punctate structures in the cytoplasm, as indicated with arrows (Figure 3A). Undoubtedly, no fluorescence signal of His protein was detected in the baculovirus uninfected Sf9 cells (Figure 3A).

TRPML is an endolysosomal calcium channel and a key player in the endolysosomal pathway [28]. To examine the localization of Ls-TRPML in more detail, we explored the localization relations of Ls-TRPML with lysosome in Sf9 cells; we found that LysoTracker, the indicator of lysosome, was localized in the cytoplasm and formed punctate structures (presented as green) in the control treated Sf9 cells (indicated with arrows), while Ls-TRPML (presented as red) mainly co-localized with LysoTracker (presented as green) in the cytoplasm of Ls-TRPML-His baculovirus-infected Sf9 cells (Figure 3B). Thus, these results revealed the characterization and localization of Ls-TRPML further.

### 3.4. The Subcellular Localization of Ls-TRPML in L. striatellus

**Figure 4:**
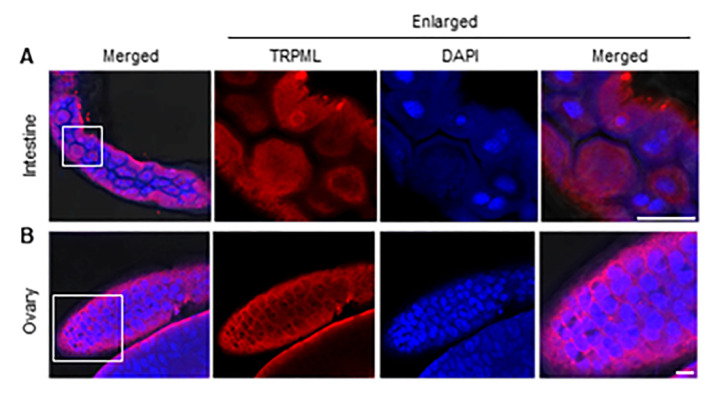
Subcellular localization of Ls-TRPML in *L. striatellus*. (**A**) Localization of Ls-TRPML in *L*. *striatellus* intestine. (**B**) Localization of Ls-TRPML in *L*. *striatellus* ovary. Organs were dissected, fixed and immunolabeled with Ls-TRPML polypeptide antibody and dyed with DAPI, and then analyzed with confocal microscopy. Scale bars, 15 μm.

The TRPML channels are the only cation channels known so far to be localized and function in the cellular digestive tract [5]. The intestine of the insect is a pivotal organ for studying genes characterization. To investigate the localization of Ls-TRPML in real condition, we dissected and immunolabeled the intestine of adult *L. striatellus* and found that Ls-TRPML polypeptide antibody could be specifically detected in the epithelial cells of the intestine, the Ls-TRPML located in the cytoplasm and some of the Ls-TRPML located around the nuclei of *L. striatellus* cells (Figure 4A). Due to the high expression of Ls-TRPML in the ovary, we also dissected and immunolabeled the ovary with Ls-TRPML polypeptide antibody. The Ls-TRPML specifically localized in the cytosol of follicular cells (Figure 4B), suggesting the potential role of Ls-TRPML in female *L. striatellus*.

### 3.5. The Specific Binding of Ls-TRPML with PI(3,5)P_2_

**Figure 5:**
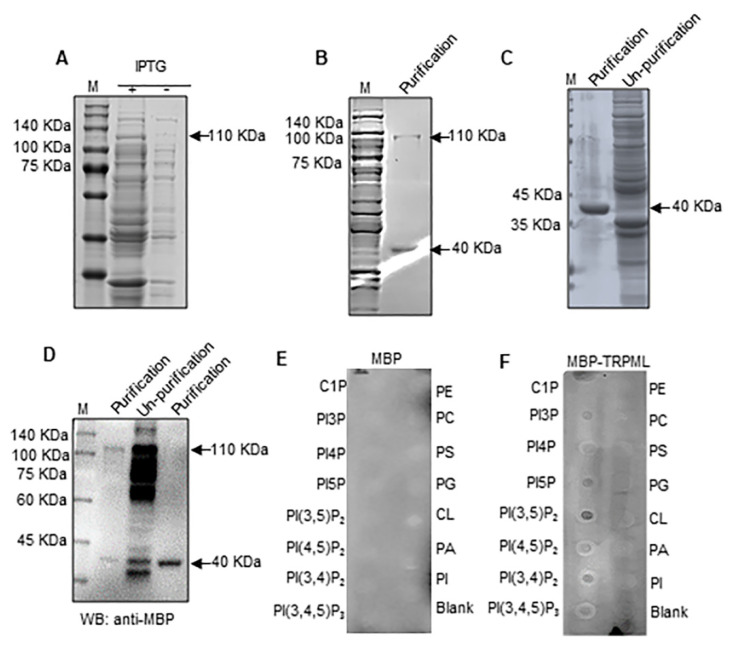
Binding of Ls-TRPML with PI(3,5)P_2_. (**A**) Induction and expression of MBP-Ls-TRPML in *E*. *coli* cells. (**B**) Purification and detection of MBP-Ls-TRPML by Coomassie brilliant blue R250. (**C**) Purification and detection of MBP by Coomassie brilliant blue R250. (**D**) Purified proteins were detected by Western blotting with MBP specific antibody. (**E**) Binding between MBP with phospholipids was introduced as control; (**F**) binding of MBP-Ls-TRPML and phospholipids was analyzed by lipid binding assay. Arrows indicate the targeted protein; +, induction with IPTG; −, control; M, marker; LPA, lysophosphatidic acid; LPC, lysophosphatidylcholine; S1P, sphingosine 1-phosphate; C1P, ceramide-1-phosphate; PI3P, phosphatidylinositol-3-phosphate; PI4P, phosphatidylinositol-4-phosphate; PI5P, phosphatidylinositol-5-phosphate; PI(3,5)P_2_, phosphatidylinositol-3,5-bisphosphate; PI(4,5)P_2_, phosphatidylinositol-4,5-bisphosphate; PI(3,4)P_2_, phosphatidylinositol-3,4-bisphosphate; PI(3,4,5)P_3_, phosphatidylinositol-3,4,5-triphosphate; PE, phosphatidylethanolamine; PC, phosphatidylcholine; PS, phosphatidylserine; PG, phosphatidylglycerol; CL, cardiolipin; PA, phosphatidic acid; PI, phosphatidylinositol.

TRPML1 is activated by phosphatidylinositol-3,5-biphosphate [PI(3,5)P_2_] [12,29,30,31]. To figure out whether Ls-TRPML interacts with PI(3,5)P_2_, we cloned the open reading frame of Ls-TRPML by RT-PCR, inserted the sequence into the pMal-c2X vector and verified the positive construct by sequencing and double digestion (Figure S3). To obtained the purified protein of Ls-TRPML to examine the binding of Ls-TRPML with PI(3,5)P_2_, we expressed the pMal-c2X-Ls-TRPML in *Rossetta* (DE3) *E. coli* cells by IPTG induction, and got a specific ~110 KDa protein with IPTG addition comparing with the control treatment (Figure 5A). The MBP-Ls-TRPML protein and MBP protein were purified according to the manufacturer’s instructions and detected by SDS–PAGE stained with Coomassie brilliant blue R250 (Figure 5B,C); they were then validated by Western blotting (Figure 5D). We used the purified MBP protein (control) or purified MBP-Ls-TRPML protein to incubated the lipid strip, respectively, by lipid binding assay according to the manufactural instruction. The results showed that the MBP-Ls-TRPML protein bound to PIPs, PI3P, PI4P, PI5P, PI(3,5)P_2_, PI(4,5)P_2_, PI(3,4)P_2_, PI(3,4,5)P_3_, with the highest affinity to PI(3,5)P_2_, while the MBP protein alone could not bind to any PIPs (Figure 5E,F), indicating that the specific binding of MBP-Ls-TRPML with PI(3,5)P_2_ was mediated by TRPML protein.

## 4. Discussion

Although the function of TRPML has been studied extensively in flies and worms, the TRPML function in agricultural pests remains unknown. In contrast, TRPML1 has six transmembrane segments and a pore loop between transmembrane segments [1], and Ls-TRPML has five transmembrane regions from 304–522 aa predicted by Smart online website (Figure S1A,C). However, protein-domain prediction by InterPro showed that Ls-TRPML contains one transmembrane region and one polycystic kidney disease (PKD) channel (Figure S1A). The PKD channel was related to TRPP, the unclassical TRPs [32]. We analyzed the identity of Ls-TRPML with Ls-TRPP-like protein (RZF41628.1) and found that the two proteins only share 8.27% of identity (Figure S4). However, whether Ls-TRPML has a TRPP function in cellular processes in *L. striatellus* needs further exploration. The different predictions between InterPro and Smart may be caused by the database or algorithm difference referred by the website. The TRPML of different agricultural insect pests is evolutionarily conserved (Figure 1A), and Ls-TRPML shares 92.41% of its amino acid identity with the Nl-TRPML of another rice planthopper; whether the TRPMLs shares similar functions in agricultural pests needs to be amply investigated in the future.

One notable sequence motif in Ls-TRPML is a typical bipartite nuclear localization signal, as with other TRPMLs [33]. We observed that Ls-TRPML-His localized in the nuclear membrane and some aggregated in the nuclei of Sf9 cells (Figure 3A); whether the nucleus entry Ls-TRPML phosphorylated to modulate other proteins functions in *L. striatellus* needs to be explored further. Consistent with the localization of *Drosophila* TRPML in HEK293 and *Drosophila* cells [7,34], Ls-TRPML-His localized in the plansma membrane, nuclear membrane and nucleus and co-localized with lysosome in Sf9 cells (Figure 3A,B). The specific polypeptide antibody was used to detect the localization by immunofluorescence microscopy, and we found that Ls-TRPML was located around the nuclei in *L. striatellus* intestine and follicular cells (Figure 4A,B). The results are consistent with the localization of Ls-TRPML-His in Sf9 cells. The localization to the plasma membrane and lysosome suggests that Ls-TRPML may be involved in mediating membrane and endolysosome trafficking.

Ls-TRPML is ubiquitously expressed among different organs. The transcript and protein level of Ls-TRPML in the ovary were notably higher than that in other organs, resulting the high expression in female adult *L. striatellus* (Figure 2A,B). TRP channels, as the calcium permeable cation channel, exert their role as sensory detectors in both male and female gametes and play regulatory functions in germ cell development and maturation [35]. The Ca^2+^ is taken up and stored in the endoplasmic reticulum (ER) to be utilized during oocyte maturation and fertilization [36,37]. What is more, the requirement of Ca^2+^ for egg activation and ovulation is evolutionarily conserved and found throughout the animal kingdom, from arthropods to higher primates [35,38]. Whether Ls-TRPML responds to a variety of signals to change the Ca^2+^ waves during oocyte maturation and fertilization and then modulates the reproduction needs further investigation.

TRPML, as a key regulator, could be activated by PI(3,5)P_2_ and manipulates Ca^2+^ release to regulate the autophagy pathway [7,34,39]. In this study, we found that Ls-TRPML was bound to PI(3,5)P_2_ stronger than with other PIPs by lipid-binding assay (Figure 5E), indicating that PI(3,5)P_2_, the low-abundance PIPs [40], may play a role in manipulating autophagy biogenesis. Viral infection triggers autophagy response in vector insect [41,42]. As *L. striatellus* is the vector of various crops viruses, whether Ls-TRPML interacts with or is hijacked by other host factors to modulate the autophagy pathway to regulate the viral infection needs to be further explored by detailed studies. In non-immune cells, the TRPML2 channel, as one of the interferon stimulating genes, enhances yellow fever virus infectivity by promoting viral uptake [43,44,45], while in immune cells, the higher expression of TRPML2 directly improves and activates PAMP receptor engagement to clear the virus [46,47,48]. Whether viral infection increased Ls-TRPML expression to arouse or inhibit the immune response pathway to regulate viral infection in *L. striatellus* needs further exploration. Such research studies will not only advance our knowledge of fundamental processes of Ls-TRPML involved in cell biology, but also will help us to elucidate the TRPML function in pathogenesis modulated by pathogenic microbes.

## 5. Conclusions

Here, in this study, we identified and characterized the TRPML protein of one agricultural pest, *Laodelphax striatellus* (small brown planthopper). We first analyzed the evolutionary tree of TRPML proteins of agricultural pests and found a close evolutionary relationship between Ls-TRPML and *Nilaparvata lugens* (brown planthopper). Next, we examined and found that Ls-TRPML is predominately expressed in the ovary of *L. striatellus* at the transcript and protein levels. Subsequently, we revealed the subcellular localization of Ls-TRPML protein in *Spodoptera frugiperda* cells and *L. striatellus* organs by the Bac–Bac system and immunofluorescence microscopy, respectively. At last, we elucidated the interaction of Ls-TRPML with phosphoinositides by lipid-binding assay. The high expression of Ls-TRPML in the ovary deserves further specific studies to evaluate the potential role in the ovary development or reproduction of *L. striatellus*. Moreover, the specific binding of Ls-TRPML with phosphatidylinositol-3,5-bisphosphate [PI(3,5)P_2_] suggests the complex role of Ls-TRPML in multiple cellular process.

## Data Availability

Not applicable.

## Supplementary Materials

The following are available online at https://www.mdpi.com/article/10.3390/insects12121107/s1. Figure S1: Functional analysis of Ls-TRPML. (**A**) Functional modules of Ls-TRPML analyzed by Smart website and cNLS website. (**B**) Amplification of Ls-TRPML by RT-PCR. (**C**) Functional domain of Ls-TRPML was predicted by Smart website. (**D**) Functional domain analysis of Ls-TRPML by InterPro online website (http://www.ebi.ac.uk/interpro/, accessed on 11 December 2021). Figure S2: Detection of Ls-TRPML polypeptide antibody by Western blotting. Arrow indicates the desired protein. Figure S3: Double digestion detection of Pmal-c2X-TRPML. Figure S4: Protein-sequence identity of Ls-TRPML and Ls-TRPP-like protein. Table S1: Primers used in the research. Sequence in lowercase is the vector sequence. Underlined sequence is 6×His-tag sequence. Red color sequence is target gene sequence.
