# Supplementary figures and images for: Identification and Characterization Analysis of Transient Receptor Potential Mucolipin Protein of Laodelphax striatellus Fallén
Source: Insects. 2021 Dec 12;12(12):1107. doi: 10.3390/insects12121107 (PMC8706664; doi:10.3390/insects12121107)

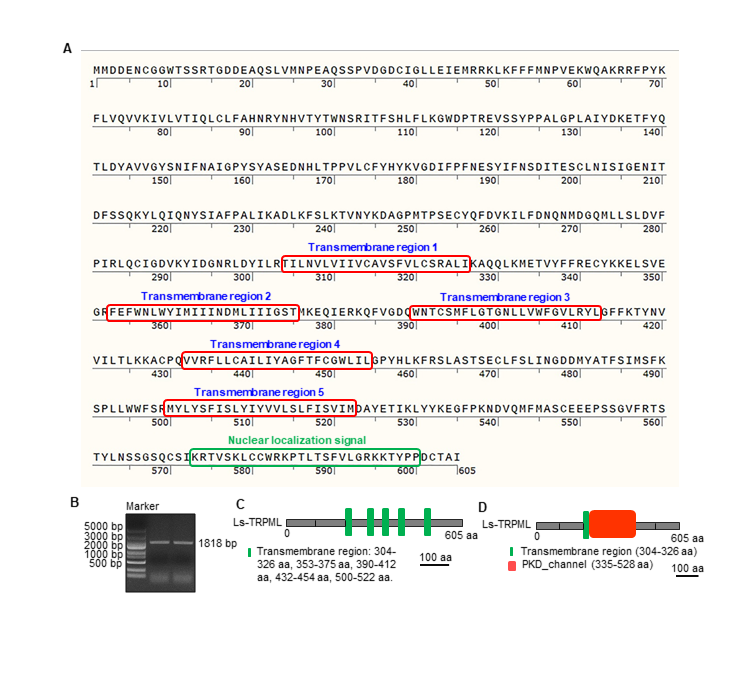

Supplement: Supplementary file 1 [file insects-12-01107-s001.zip › Figure S1.tif]

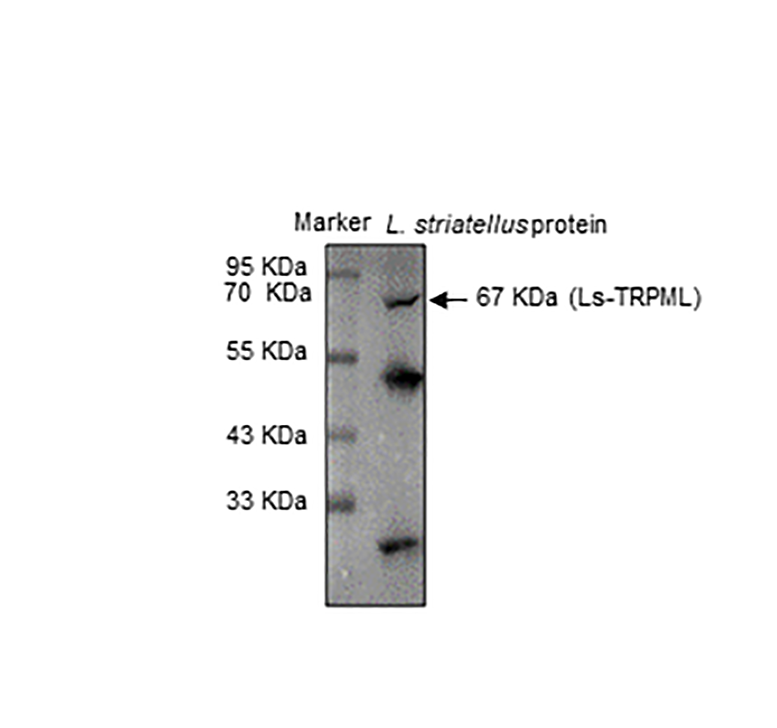

Supplement: Supplementary file 1 [file insects-12-01107-s001.zip › Figure S2.tif]

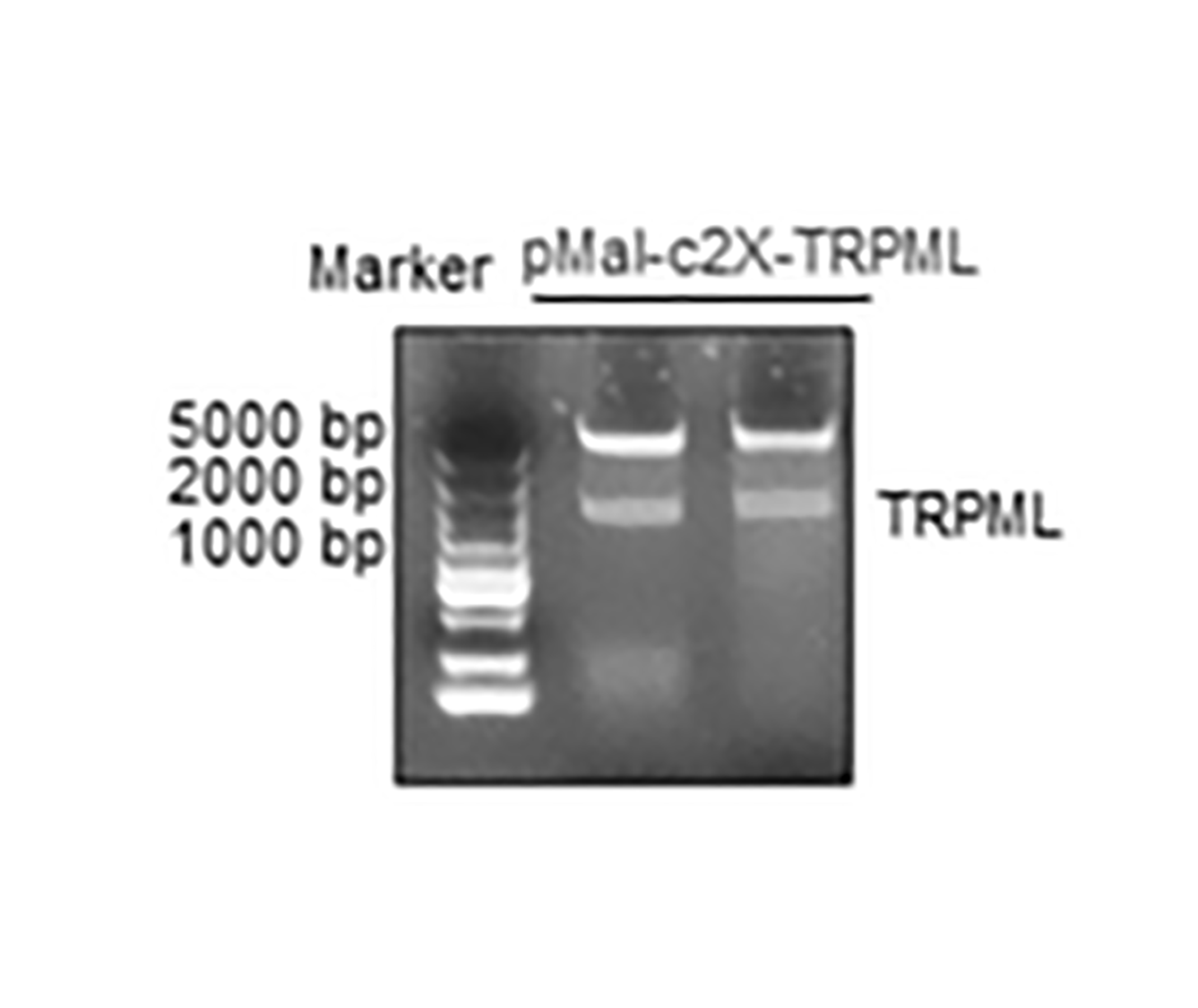

Supplement: Supplementary file 1 [file insects-12-01107-s001.zip › Figure S3.tif]

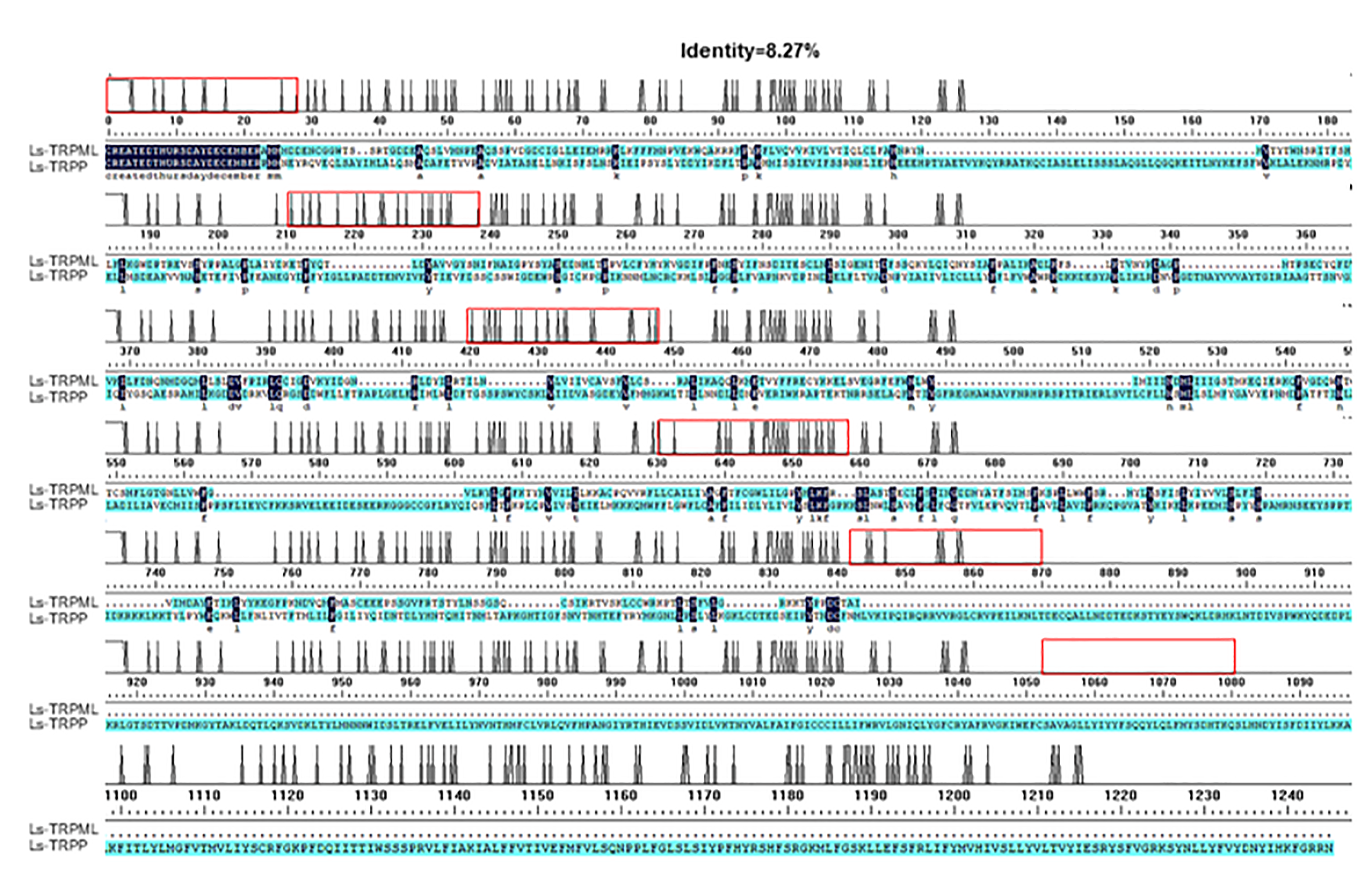

Supplement: Supplementary file 1 [file insects-12-01107-s001.zip › Figure S4.tif]
